# Allelic and Genotype Frequencies of CYP2B6^∗^2 (64C > T) and CYP2B6^∗^3 (777C > A) in Three Dominant Ethnicities of the Iranian Population

**DOI:** 10.1155/2023/8283470

**Published:** 2023-02-09

**Authors:** Armin Khavandegar, Bahareh Tavakoli-Far, Sarina Ansari, Parisa Veis-Karami, Faezeh Ghasemi, Samira Sheibaninia, Roshanak Jazayeri, Massoud Houshmand

**Affiliations:** ^1^Student Research Committee, Alborz University of Medical Science, Karaj, Iran; ^2^Dietary Supplements and Probiotic Research Center, Alborz University of Medical Sciences, Karaj, Iran; ^3^Department of Physiology and Pharmacology, School of Medicine, Alborz University of Medical Sciences, Karaj, Iran; ^4^Department of Medical Genetics, National Institute for Genetic Engineering and Biotechnology (NIGEB), Tehran, Iran; ^5^Department of Genetics, Faculty of Medicine, Alborz University of Medical Sciences, Karaj, Iran

## Abstract

**Background:**

Cytochrome P450 complex plays a key role in drug metabolism. CYP2B6 has an essential part in Cytochrome P450 complex metabolism. This study aims to determine the allelic distribution of CYP2B6^∗^2 and CYP2B6^∗^3 in three main Iranian ethnicities: Fars, Turk, and Kurd.

**Methods:**

The study was conducted on 174 unrelated healthy volunteers from three main Iranian ethnicities. After DNA extraction from peripheral blood samples, genotyping of CYP2B6^∗^2 and ^∗^3 was performed using tetra ARMS and ARMS PCR, respectively.

**Results:**

The average age of 174 cases was 40.69 ± 11.87 (mean ± SD) and 39.06 ± 11.63 (mean ± SD) for males and females. In the CYP2B6^∗^2 variant, the genotyping frequency of wild type (C/C), heterozygous (C/T), and homozygous mutant (T/T) was 8.7%, 86%, and 5.2%, respectively. The CYP2B6^∗^2 (c.64C > T) allele frequency was 48.2% (95% CI: (37.8–58.6)). In the CYP2B6^∗^3 variant, the frequency of wild type (C/C), heterozygous (C/T), and homozygous mutant (T/T) was 75.3%, 11%, and 13.6%, respectively. The CYP2B6^∗^3 (c.777C > A) allelic frequency was 19.1% (95% CI: (17.5–20.7)).

**Conclusion:**

Allelic distribution in three main Iranian ethnicities, i.e., Turk, Kurd, and Fars, is remarkably higher than that in other populations, even that in Southern Iran. High frequencies of CYP2B6^∗^2 and ^∗^3 in the Iranian population highly affect drug responsiveness. Understanding such variability could help to increase drug efficacy and reduce its toxicity.

## 1. Introduction

Personalized medicine addresses the terms covered by targeted therapies, related diagnostics, and the medical intervention's safety [1]. Following the evolution of the Human Genome Project, the association between personalized medicine and genes encoding drug-metabolizing proteins became more outstanding [2]. Amongst all those proteins, Cytochrome P450, known as CYP, is responsible for the metabolization of roughly 90% of currently prescribed medications, apart from its intrinsic activities [3].

Cytochrome P450 enzymes play a crucial role in the metabolism of different agents, including food and drugs, with various enzymatic activities leading to various drug responses among individuals [4]. Many highly polymorphic genes encode cytochrome P450, and several Single Nucleotide Polymorphisms (SNPs) have been identified in each gene [5]. SNPs may result in various enzymatic activities in people, eventually leading to insufficient efficacy of medical regimens or their adverse effects in a constant amount of drugs [6].

Ethnicity is among those factors influencing the SNP frequencies and various drug responses [7]. Although there are worldwide databases providing the frequency of multiple SNPs, they still need some data on specific ethnicities. Based on the latest studies, Iran's population was calculated at 83.9 million in 2020 and accounted for the 18^th^ most populated country in the world [8]. Based on available data, 65% of the Iranian population are Fars, 16% Turk, 7% Kurd, 6% Lor, and 6% other ethnicities. In other words, Fars, Turk, and Kurd together account for 88% of the Iranian population [9].

CYP2B6 belongs to the Cytochromes P450 (CYPs) superfamily of enzymes that are essential for the clearance of various compounds, as well as for hormone synthesis [10, 11]. CYP2B6 participates in the metabolism of drugs, including many antibiotics, antiretroviral drugs, antimalarial, and first-lineanti-Tuberculosis drugs [12]. CYP2B6 is polymorphic and contains significant interindividual variability in the human population [13]. Hence, the analysis of CYP2B6 SNPs may improve drug efficacy and also decrease medical regimens' adverse effects.

Based on Lang et. al, CYP2B6 primarily consisted of 9 SNPs in 6 allelic variations; CYP2B6 c.64C > T as ^∗^2, CYP2B6 c.777C > A as ^∗^3, CYP2B6 c.785A > G as ^∗^4, CYP2B6 c.1459C > T as ^∗^5, CYP2B6 c.516G > T and c.785A > G as ^∗^6, and CYP2B6 c.516G > T and c.785A > G and c.1459C > T as ^∗^7 [14]. To date, the last CYP2B6 constellation is named ^∗^38 [15].

Studies on the CYP2B6 isozyme have expanded significantly since 2003, at the same time as the discovery of this isozyme effect in the clearance of the antiviral drug, especially efavirenz [13]. Besides, in another study conducted in 2003, the clearance of bupropion drug in people with the CYP2B6^∗^4 variant was 1.6 times more than in patients of other variants [16]. A higher concentration of anti-HIV drugs, including efavirenz, and its toxicity in CYP2B6^∗^2 [17] have been reported.

Various drugs are metabolized by CYP2B6 [18, 19]; among them, two drugs that are more specifically metabolized by a CYP2B6 isoenzyme are methadone and bupropion, and these two drugs are even utilized as probes to detect the function and investigate the behavior of the CYP2B6 enzyme [20, 21]. Reports show that Iran has a considerable rate of opium use, especially methadone [22]. To the extent of our knowledge, CYP2B6^∗^2 and ^∗^3 have not been evaluated in Iranian populations, except for Southern Iran [23], which is previously reported to have a distinguished genetic pool from other Iranian populations [24].

In this study, due to the scarcity of data on the distribution of CYP2B6 c.64C > T (rs8192709) and CYP2B6 c.777C > A (rs45482602) in the Iranian population, we aimed to evaluate the mentioned polymorphism in three dominant Iranian ethnicities, Fars, Turk, and Kurd.

## 2. Methods and Materials

### 2.1. Samples Collection and Ethical Consideration

One hundred and seventy-four samples from unrelated healthy donors, aged 18–60, of three ethnicities of the Iranian population from various provinces, were achieved. Patients with a history of cancer, metabolic disorders, and any disease affecting DNA were excluded from the study. Of this number, 65 (37.4%) cases were males and 109 (62.6%) were females. The average age of cases was 40.69 ± 11.87 (mean ± SD) and 39.06 ± 11.63 (mean ± SD) for males and females, respectively. Two milliliters of blood samples encompassing 80 Fars, 69 Turk, and 25 Kurd were collected in ethylenediaminetetraacetic acid (EDTA) enriched tubes. An informed written consent form was obtained from each person to permit genetic analysis and publication. Besides, the Medical ethics committee has approved the study.

### 2.2. DNA Extraction and Primer Sequencing

DNA extraction was performed using an MBST kit (salting-out method) in two milliliters of blood samples. For DNA isolation, lysis buffer for the digestion of non-nucleic acid components of the cell, precipitation buffer for protein isolation, washing buffer, and eventually elusion buffer for DNA resuspension were utilized. Therefore, we utilized Oligonucleotide F (forward) and R (reverse) primers for CYP2B6 ^∗^2 and ^∗^3 amplification, as shown in Table 1.

### 2.3. ARMS and Tetra ARMS-PCR

**Figure 1:**
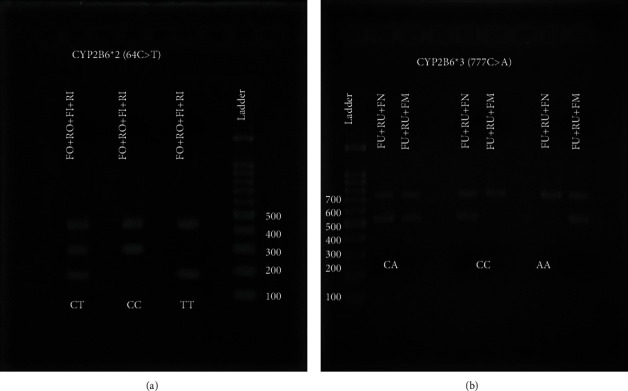
(a) A heterozygous, wild type, and homozygous mutant, left to right, in rs8192709 in three cases after tetra ARMS-PCR. (b) A heterozygous, wild type, and homozygous mutant, left to right, in rs45482602 in three cases after ARMS-PCR.

Genotyping analysis was conducted utilizing an amplification refractory mutation system polymerase chain reaction (ARMS PCR) for CYP2B6^∗^3 and tetra ARMS PCR for CYP2B6^∗^2. The total volume of each PCR reaction was 25 microliters consisting of 0.6 *μ*l of each F and R primers, two *μ*l DNA template, and 11 *μ*l EmeraldAmp PCR master mix. The PCR cycling was set at 96°C for 4 minutes, followed by 30 cycles of denaturation at 95°C for 30 seconds, annealing for 45 seconds, extension at 72°C for 45 seconds, and final extension at 72°C for 8 minutes. The 3% agarose gel was used to rub the PCR products. Eventually, visualization was performed by staining with ethidium bromide and analyzed by the gel documentation system (Figure 1).

### 2.4. Sequencing Analysis

Similar to our previous studies [25], forwards and reverse primers sequenced PCR products on an automated ABI 3100 sequencing machine (Applied Biosystems, Kavosh Fanavaran Kawsar Company, Iran). Then, we used the Finch TV program for sequencing and analyzing to confirm the results of nucleotide variations.

### 2.5. Statistical Analysis

The statistical analyses were performed using SPSS (version 24; IBM) software. A confidence interval test (95%) was considered for the frequency of alleles and genotypes. A *P* value of less than 0.05 was considered statistically significant [25].

## 3. Results

### 3.1. Demographic Distribution

In this study, 174 samples of whole blood cells were collected from three dominant Iranian ethnicities consisting of 80 (46%) Fars, 69 (39.7%) Turk, and 25 (14.4%) Kurd to investigate the allelic and phenotypic distribution of CYP2B6^∗^2 and ^∗^3. There was no significant correlation between the samples' gender and allelic genotypes.

### 3.2. CYP2B6 (c.64 > T) Allelic and Genotype Frequency

CYP2B6 wild-type homozygote (C/C) was 87% (95% CI (6.7–10.7)), while mutated homozygote (T/T) and heterozygote (C/T) genotypes were 5.2% (95% CI (3.9–6.5)) and 86% (95% CI (83.2–88.8)), respectively. C/C genotype highest and lowest frequencies were observed in Kurd (12% (95% CI (10.3–13.7))) and Fars (6.3% (95% CI (5.7–6.9))), respectively. T/T genotype was highest in Turk (10.3% (95% CI (5.2–15.4))) and lowest in Kurd (0%). Furthermore, C/T genotype ranged from 91.1% (95% CI (87.5–94.7)) in Fars to 79.4% (95% CI (73.5–85.5)) in Turk. The CYP2B6^∗^2 (c.64 C > T) allele was 48.2% (95% CI (37.8–58.6)), ranging from 44% (95% CI (36.8–51.2)) in Kurd to 50% (95% CI (41.6–58.4)) in Turk. Tables 2 and 3 summarize the genotype and allelic frequency, respectively.

### 3.3. CYP2B6^∗^3 (c.777C > A) Allelic and Genotype Frequency

CYP2B6 wild-type homozygote (C/C) was 75.3% (95% CI (70.1–80.5)), while mutated homozygote (A/A) and heterozygote (C/A) genotypes were 13.6% (95% CI (12.5–14.7)) and 11% (95% CI (8.3–13.7)), respectively. C/C genotype highest and lowest frequencies were observed in Turk (77.8% (95% CI (74.6–81))) and Kurd (72% (95% CI (65.3–78.7))), respectively. A/A genotype was highest in Fars (16% (95% CI (13.7–18.3))) and lowest in Turk (11.1% (955 CI (7.4–14.8))). Furthermore, C/A genotype ranged from 9.3% (95% CI (7.2–11.4)) in Fars to 16% (95% CI (11.8–20.2)) in Kurd. The CYP2B6^∗^3 (c.777C > A) allele was 19.1% (95% CI (17.5–20.7)), ranging from 16.6% (95% CI(12.8–20.4)) in Turk to 20.6 (95% CI (16.2–25)) in Fars. Tables 2 and 3 summarize the genotype and allelic frequency, respectively.

## 4. Discussion

CYP2B6 plays a crucial role in the metabolism of antiretroviral drugs, including efavirenz, immunosuppressant drugs, including cyclophosphamide, antidepressants, e.g., bupropion, antiepileptics, e.g., valproic acid, and antiarrhythmic, e.g., mexiletine, among other drugs, by changing to their metabolic forms [13, 26–29]. Also, it is believed that CYP2B6 expression is higher in smokers and alcoholics compared to the normal population [30].

To the extent of our knowledge, this is the first study investigating the frequency of CYP2B6^∗^2 and CYP2B6^∗^3 in the three main Iranian populations: Fars, Turk, and Kurd. In this study, CYP2B6^∗^2 frequency was 48.2%, ranging from 44% in Kurd to 50% in Turk ethnicities; besides, CYP2B6^∗^3 frequency was 19.1% on average, ranging from 16.6% in Turk to 20% in Kurd. In a survey in Japan by Hiratsuka et al., CYP2B6^∗^2 and CYP2B6^∗^3 frequencies were 4.7% and 0%, respectively; besides, the same alleles frequencies in the Caucasian population were reported at 5.3% and 0.5%, respectively [31].

In another study evaluating the CYP2B6 allelic distribution in 631 West African populations, the frequency of CYP2B6^∗^3 was 0%, while the frequency of CYP2B6^∗^2 was 2.82% [32]. In a study by Musa et al., CYP2B6^∗^2 allelic distribution was 0.8% in Malaysian and 4.1% in Indians [33]. Another study investigating the allelic frequency of CYP2B6 in ethnicity in Southern Iran, known as the Baluch population, reported a 3.9% allelic distribution of CYP2B6^∗^2 [23]. In a study in 2021, CYP2B6^∗^3 allelic distribution was reported at 6.5% of the Pakistan population [34].

As demonstrated in Tables 4 and 5, the CYP2B6^∗^2 and ^∗^3 allelic frequency is remarkably higher in the three main Iranian populations than in other ethnicities, even those in Southern Iran. It is believed that due to a considerable number of consanguineous marriages in Iran, autosomal recessive diseases highly manifest in this country; furthermore, it is said that Iranian allele frequency is noticeably different from those in Europeans, while Iranian Baluch seemed to have a tendency towards south Asians in allele frequency [23, 24]. Besides, CYP2B is believed to have the most frequent interpersonal variety, which accounted for 20–250 times more than other Cytochrome P450 isoenzymes [40].

Our results, alongside those from other studies, provide further evidence for ethnic heterogeneity in drug metabolism. We hope our findings contribute to a better understanding of various drug responses in different populations.

## 5. Study Limitations

This study was conducted on 173 cases from three various major Iranian ethnicities. Further studies are needed to evaluate the allelic polymorphism in larger populations and other ethnicities.

## 6. Conclusion

Allelic distribution in three main Iranian ethnicities, i.e., Turk, Kurd, and Fars, is remarkably different from other populations, even those in Southern Iran. High frequencies of CYP2B6^∗^2 and ^∗^3 in the Iranian population remarkably affect drug responsiveness, including higher efavirenz toxicity. Understanding such variability could help to increase drug efficacy and reduce its toxicity.

## Figures and Tables

**Table 1:** Oligonucleotide sequences of primers in real-time, rapid-cycle PCR.

SNP	Primer	Sequence	Position	PCR technique
CYP2B6 c.64C > T (p.R22 > C) or rs8192709 or^∗^2	FO	5′GGGATAGGCATCAGGTCACTGG3′	FO-RO 431	Tetra ARMS
	RO	5′TTCCCCAAGTACCAAGGCAAGA3′	FO-RI 283	
	FI	5′CTCTTGCTACTCCTGGTTCAGT3′	FI-RO 189	
	RI	5′TCATGGGTGTTAGGGTGGCG3′		

CYP2B6 c.777C > A (p.S259 > R) or rs45482602 or^∗^3	FU	5′GTTCCCATGGAGGGATTGGG3′	FU-RU 734	ARMS
	RU	5′CTCTACACATCCAACCGCGTA3′	FN-RU 525	
	FM	5′ GAAACCCTGGACCCCAGA 3′	FM-RU 525	
	FN	5′ GAAACCCTGGACCCCAGC3′		

**Table 2:** Analysis of the CYP2B6^∗^2 allelic frequencies (%) among three dominant ethnicities in Iran; the correlation between ethnicity genotype frequency ethnicity and the each ethnicity is shown.

Number (%)		Ethnicity			
		Fars	Turk	Kurd	Total
		80 (46%)	69 (40%)	25 (14%)	174 (100%)
CYP2B6^∗^2	Allele C% (95% CI)	52 (47.8–56.2)	50 (47.4–52.6)	56 (49.2–62.2)	51.8 (47.1–56.5)
	Allele T% (95% CI)	48 (44.6–51.4)	50 (41.6–58.4)	44 (36.8–51.2)	48.2 (37.8–58.6)
	*P* value	0.179	0.401	0.386	

CYP2B6^∗^3	Allele C% (95% CI)	79.4 (71.6–87.2)	83.4 (70–96.8)	80 (74.4–85.6)	80.9 (74.1–87.7)
	Allele A% (95% CI)	20.6 (16.2–25)	16.6 (12.8–20.4)	20 (17.8–22.2)	19.1 (17.5–20.7)
	*P* value	0.609	0.796	0.681	

**Table 3:** Analysis of the CYP2B6^∗^2 genotypic frequencies (%) among three dominant ethnicities in Iran; the correlation between ethnicity genotype frequency ethnicity and the each ethnicity is shown.

Number (%)		Ethnicity			
		Fars	Turk	Kurd	Total
		80 (46%)	69 (40%)	25 (14%)	174 (100%)
CYP2B6^∗^2	Wild (C/C) (95% CI)	6.3% (5.7–6.9)	10.3% (9.3–11.3)	12% (10.3–13.7)	8.7% (6.7–10.7)
	Heterozygous (C/T) (95% CI)	91.1% (87.5–94.7)	79.4% (73.5–85.5)	88% (80.2–95.8)	86% (83.2–88.8)
	Mutant (T/T) (95% CI)	2.5% (1.7–3.3)	10.3% (5.2–15.4)	0%	5.2% (3.9–6.5)
	*P* value	<0.001^*∗∗*^	0.041^*∗*^	<0.001^*∗∗*^	

CYP2B6^∗^3	Wild (C/C) (95% CI)	74.7% (70.6–78.8)	77.8% (74.6–81)	72% (65.3–78.7)	75.3% (70.1–80.5)
	Heterozygous (C/A) (95% CI)	9.3% (7.2–11.4)	11.1% (6.6–15.6)	16% (11.8–20.2)	11% (8.3–13.7)
	Mutant (A/A) (95% CI)	16% (13.7–18.3)	11.1% (7.4–14.8)	12% (9.1–14.9)	13.6% (12.5–14.7)
	*P* value	<0.001^*∗∗*^	<0.001^*∗∗*^	<0.001^*∗∗*^	

^*∗*^ *P* value of less than 0.05 is statistically significant. ^*∗∗*^*P* value of less than 0.01 is statistically highly significant.

**Table 4:** Comparison of allele frequency of CYP2B6^∗^2 reported in various populations.

Authors	Year	Population	^∗^2 frequency (%)	Heterozygous	Sample size	Reference
				Homozygous		
Current study	2022	Iranian	48.2	86%	174	
				5.2		

Thomas Lang	2001	Caucasian	5.4	10.7%	215	[14]
				0		

Hiratsuka et al.	2002	Japanese	4.7	N/A	530	[31]
				N/A		

Hiratsuka et al.	2002	Caucasian	5.3	N/A	430	[31]
				N/A		

Mehlotra et al.	2006	West Africa	2.82	N/A	631	[32]
				N/A		

Musa et al.	2012	Malaysia	0.8	N/A	392	[33]
				N/A		

Musa et al.	2012	Chinese	1.3	N/A	330	[33]
				N/A		

Musa et al.	2012	Indians	4.1	N/A	126	[33]
				N/A		

Zakeri et al.	2014	Southern Iran	3.9	N/A	206	[23]
				N/A		

Tomas et al.	2017	Croatian Roma	12.8	22.9%	436	[35]
				1.4%		

Cho et al.	2004	Korean	3	N/A	358	[36]
				N/A		

Lamba et al.	2003	Hispanics	14	N/A	14	[37]
				N/A		

Lamba et al.	2003	Caucasians	9	N/A	86	[37]
				N/A		

Carano et al.	2018	Italian	6.61	N/A	348	[38]
				N/A		

Arnaldo et al.	2013	Mozambican	5.7	7.8%	360	[39]
				1.9%		

**Table 5:** Comparison of allele frequency of CYP2B6^∗^3 reported in various populations.

Authors	year	Sample size	^∗^3 frequency (%)	Heterozygous	Sample size	Reference
				Homozygous		
Current study	2022	Iranian	19.1	11%	174	
				13.6%		

Thomas Lang	2001	Caucasian	0.5	0.9%	215	[14]
				0		

Hiratsuka et al.	2002	Japanese	0	N/A	530	[31]
				N/A		

Hiratsuka et al.	2002	Caucasian	0.5	N/A	430	[31]
				N/A		

Mehlotra et al.	2006	West Africa	0	N/A	631	[32]
				N/A		

Ahmed et al.	2021	Pakistan	6.5	6.73%	490	[34]
				3.06%		

Cho et al.	2004	Korean	0	0	358	[36]
				0		

Arnaldo et al.	2013	Mozambican	0	0	360	[39]
				0		

## Data Availability

We have presented all the necessary data in this manuscript. The data would be available from the corresponding author on reasonable request.
